# Supplementary material for: What does it take to learn the rules of RNA base pairing? A lot less than you may think
Source: bioRxiv. 2025 Aug 2:2025.07.31.668042. Preprint. [Version 1] doi: 10.1101/2025.07.31.668042 (PMC12324431; doi:10.1101/2025.07.31.668042)
Supplement: Supplement 1 [file media-1.gz › supplemental_material/src/rscape_v2.6.0.tar.gz!/rscape_v2.6.0/lib/infernal/Manuscripts/banded-cyk/main.doc]

Query-Dependent Banding (QDB) for Faster RNA Similarity Searches

Eric P. Nawrocki and Sean R. Eddy

HHMI Janelia Farm Research Campus

19700 Helix Drive

Ashburn VA 20147

http://selab.janelia.org/

**Running head:** Faster RNA Similarity Searches

**Abstract**

When searching sequence databases for RNAs, it is desirable to score both primary sequence and RNA secondary structure similarity. Covariance models (CMs) are probabilistic models well suited for RNA similarity search applications. However, the computational complexity of CM dynamic programming alignment algorithms has limited their practical application. Here we describe an acceleration method called *query-dependent banding* (QDB), which uses the probabilistic query CM to precalculate regions of the dynamic programming lattice that have negligible probability, independently of the target database. We have implemented QDB in the freely available infernal software package. QDB reduces the average-case time complexity of CM alignment from to for a query RNA of *N* residues and a target database of *L* residues, resulting in a four-fold speedup for typical RNA queries. Combined with other improvements to infernal, including informative mixture Dirichlet priors on model parameters, benchmarks also show increased sensitivity and specificity resulting from improved parameterization.

## Non-technical summary

Database similarity searching is the sine qua non of computational molecular biology. Well-known and powerful methods exist for primary sequence searches, such as BLAST and profile hidden Markov models. However, for RNA analysis, biologists rely not only on primary sequence but also on conserved RNA secondary structure to manually align and compare RNAs, and most computational tools for identifying RNA structural homologs remain too slow for large scale use. This paper describes a new algorithm for accelerating one of the most general and powerful classes of methods for RNA sequence and structure analysis, so-called profile SCFG (stochastic context-free grammar) RNA similarity search methods. We describe this approach, called *query-dependent banding* (QDB), in the context of this and other improvements in a practical implementation, the freely-available infernal software package, the basis of the Rfam RNA family database for genome annotation. infernal is now a faster, more sensitive, and more specific software tool for identifying homologs of structural RNAs.

## Introduction

Many functional RNAs conserve a base-paired secondary structure. Conserved RNA secondary structure induces long-distance pairwise correlations in homologous RNA sequences. When performing database searches to identify homologous structural RNAs, it is desirable for RNA similarity search programs to score a combination of secondary structure and primary sequence conservation.

A variety of approaches for RNA similarity searching have been described. There are specialized programs for identifying one particular RNA family or motif, such as programs that identify tRNAs [1, 2], snoRNAs [3, 4], microRNAs [5, 6], SRP RNAs [7], and rho-independent transcription terminators [8]. There are also pattern-matching algorithms that rely on expertly designed query patterns [9]. However, the most generally useful approaches are those that take any RNA (or any multiple RNA alignment) as a query, and use an appropriate scoring system to search a sequence database and rank high-scoring similarities [10, 11], just as programs like blast do for linear sequence comparison [12].

In a general search program, one wants to score a combination of RNA sequence and structural conservation in a principled rather than *ad hoc* manner. A satisfactory solution to this problem is known, using probabilistic models called *stochastic context-free grammars* (SCFGs). SCFGs readily capture both primary sequence and (non-pseudoknotted) RNA secondary structure conservation [13, 14]. Just as hidden Markov models (HMMs) are useful for many different linear sequence modeling applications, including genefinding, multiple alignment, motif finding, and similarity search [14], SCFGs are a generally useful paradigm for probabilistic RNA sequence/structure analysis, with applications including secondary structure prediction and genefinding. A particular SCFG architecture called *covariance models* (CMs) was developed specifically for the RNA similarity search problem [15]. CMs are profile SCFGs, analogous to the use of profile HMMs in sequence analysis [15, 16]. The Rfam database of RNA families [17] is based on CM software (infernal) in much the same way that the Pfam database of protein families is based on profile HMM software (hmmer) [18, 19].

The most serious problem with using CMs has been their computational complexity. Applying standard SCFG dynamic programming alignment algorithms to the particular case of CMs results in algorithms that require $O(N^{3})$ memory and $O(LN^{3})$ time for a query of length *N* residues (or consensus alignment columns) and a target database sequence of length *L*. The memory complexity problem has essentially been solved, by extending divide-and-conquer dynamic programming methods (the Hirshberg or Myers/Miller algorithm) to the case of CMs [16], but the time complexity problem still stands.

Weinberg and Ruzzo have described several filtering methods for accelerating CM searches [20, 21, 22]. The original idea (“rigorous filters”) was to score a target sequence first by a linear sequence comparison method, using a profile HMM specially constructed from the query CM such that the profile score was provably an upper bound on the CM score; the subset of hits above threshold would then be passed for rescoring with the more expensive CM alignment algorithm [21]. Subsequently a “maximum likelihood heuristic” filter profile was developed that gives up the guarantee of recovering the same hits as the unfiltered search, but offers greater speedups [22]. For most current Rfam models, Weinberg/Ruzzo filters give about a hundred-fold speed up relative to a full CM based search at little or no cost to sensitivity and specificity. However, because these filters depend on primary sequence conservation alone, they can be relatively ineffective for RNA families that exhibit poor sequence conservation – unfortunately, precisely the RNAs that benefit the most from SCFG-based search methods. Indeed, in this respect, we are concerned that the overall performance of rigorous filters on the current Rfam database may be somewhat misleading. Rfam currently uses a crude blast-based filtering method to accelerate the CM searches used in curating the database. This step introduces a bias towards high primary sequence similarity in current Rfam alignments. As Rfam improves and incorporates more diverse structural homologs, the effectiveness of sequence-based filters will decrease. To address this worry, Weinberg and Ruzzo have also described additional heuristics (“sub-CMs” and the “store-pair” technique) that should capture more secondary structure information in the filtering process [20]. Bafna and coworkers have described further improvements to sequence filtering methods [23]. Currently, the infernal codebase includes Weinberg’s C++ implementation of rigorous filters, but not, as yet, the ML heuristic, sub-CM, or store-pair methods. All these methods are important, but it also remains important to us to identify yet more methods for accelerating CMs.

Here, we describe a method for accelerating CM searches using a banded dynamic programming (DP) strategy. In banded DP, one uses some fast method to identify a band through the DP matrix where the optimal alignment is likely to lie, and then calculates computationally expensive DP recursions only within that band. In most cases, including our approach, banded DP is a heuristic that sacrifices guaranteed alignment optimality. Banding is a standard approach in many areas of sequence analysis. Gapped blast uses banded DP to convert ungapped high-scoring pairs (HSPs) to full gapped alignments [12]. lagan and multi-lagan use banded dynamic programming (referred to as limited-area dynamic programming) to stitch together alignments between anchored sequences when aligning long genomic sequences [24]. Banding has also been applied to profile SCFGs by Michael Brown in his rnacad program by using information from a profile HMM alignment to define bands for the expensive SCFG alignment [25]. The key to developing a banded DP strategy is in deciding how the bands are identified. Usually, including all the examples just mentioned, banded DP involves performing some sort of rapid approximate sequence alignment between the query and the target.

In contrast, the method we describe here, called *query-dependent banding* (QDB), takes advantage of specific properties of CMs in order to predefine bands that are independent of any target sequence. QDB depends on the consensus secondary structure of the query, so it is complementary to acceleration methods like the Weinberg/Ruzzo filters that rely on sequence but not structure.

## Results

Briefly, the key idea is the following. Each base pair and each single-stranded residue in the query RNA is represented in a CM by a *state*. States are arranged in a tree-like structure that mirrors the secondary structure of the RNA, along with additional states to model insertions and deletions. The standard CM dynamic programming alignment algorithm works by calculating the probability that a substructure of the query rooted at state *v* aligns to a subsequence *i*..*j* in the the target sequence. The calculation is recursive, starting at the leaves of the CM (ends of hairpin loops) and subsequences of length 0, and working upwards in larger substructures of the CM, and outwards in longer and longer subsequences.

To guarantee optimality, at each *v*, the DP algorithm must score all possible *i*..*j* subsequences in the target sequence. However, most of these subsequences are obviously too long or short, when one considers the size of the query substructure under state *v*. For example, when state *v* models the closing base pair of a consensus four-base loop, only *i*..*j* subsequences of length six are likely to occur in any optimal alignment to state *v*; that is, (*j*-5,*j*) being the base pair, and (*j*-4..*j*-1) being the four bases of the hairpin loop. Likewise, the optimal subsequence aligned to the next consensus base pair in that stem is almost certainly of length eight.

Because insertions and deletions may occur in the target sequence, no subsequence length is known with certainty, but because the CM is a probabilistic model, a probability distribution for subsequence lengths under each state (including the probability of insertions and deletions) can be analytically derived from the query CM. These distributions can be used to determine a band of subsequence lengths that captures all but a negligible amount of the probability mass. A CM dynamic programming algorithm can then look not at all subsequences *i*,*j* for each state *v*, but only those *i* within a band of minimum and maximum distance relative to each *j*.

To formalize this idea, we start with a description of CMs, followed by the QDB algorithms for calculating the subsequence length distributions, using these length distributions to determine bands, and using the bands in a banded CM dynamic programming alignment algorithm. Calculation of the bands is sensitive to transition parameter estimation, so we describe infernal’s new implementation of informative Dirichlet priors for CM parameter estimation. Finally, we present results from a benchmark that suggest the sensitivity and specificity of a QDB-accelerated search is negligibly different from a non-banded search.

### Covariance models

CMs are a convention for mapping an RNA secondary structure into a tree-like, directed graph of SCFG states and state transitions (or equivalently, SCFG nonterminals and production rules). The CM is organized by a binary tree of *nodes* representing base pairs and single-stranded residues in the query’s structure. Each node contains a number of *states*, where one state represents the consensus alignment to the query, and the others represent insertions and deletions relative to the query. Figure 1 shows an example of converting a consensus structure to the guide tree of nodes, and part of the expansion of those guide tree nodes into the CM’s state graph. Here we will only concentrate on the aspects of CMs necessary to understand QDB, and a subset of our usual notation. For full details on CM construction, see [16, 26].

A guide tree consists of eight types of nodes. MATP nodes represent consensus base pairs. MATL and MATR nodes represent consensus single-stranded residues (emitted to the left or right with respect to a stem). BIF nodes represent bifurcations in the secondary structure of the family, to deal with multiple stem-loops. A ROOT node represents the start of the model. BEGL and BEGR nodes represent the beginnings of a branch on the left and right side of a bifurcation. END nodes end each branch.

The CM is composed of seven different types of states, each with a corresponding form of production rule, with notation defined as follows:

| State type | Description | Production |  |  | Emission | Transition |
| --- | --- | --- | --- | --- | --- | --- |
| P | (pair emitting) | $P\to aYb$ | 1 | 1 | $e_{v}(a,b)$ |  |
| L | (left emitting) | $L\to aY$ | 1 | 0 | $e_{v}(a)$ |  |
| R | (right emitting) | $R\to Ya$ | 0 | 1 |  |  |
| B | (bifurcation) | $B\to SS$ | 0 | 0 | 1 | 1 |
| D | (delete) | $D\to Y$ | 0 | 0 | 1 |  |
| S | (start) | $S\to Y$ | 0 | 0 | 1 |  |
| E | (end) | $E\to\varepsilon$ | 0 | 0 | 1 | 1 |

That is, for instance, if state *v* is a pair state, it produces (aligns to and scores) two correlated residues *a* and *b* and moves to some new state *Y*. The probability that it produces a residue pair *a*,*b* is given by an *emission probability* $e_{v}(a,b)$. The probability that it moves to a particular state *Y* is given by a *transition probability* $t_{v}(Y)$. The set of possible states *Y* that *v* may transit to is limited to the states in the next (lower) node in the guide tree (and insert states in the current node); the set of possible children states *Y* is called , for “children of *v*”. The indicators and are used to simplify notation in CM dynamic programming algorithms. They are the residues emitted to the left and right of state *v*, respectively. Bifurcation rules are special, in that they always transition to two particular start (S) states, at the root of subtrees in the guide tree, with probability 1.0.

These state types essentially define a “normal form” for SCFG models of RNA, akin to SCFGs in Chomsky normal form where all productions are in one of two forms, $Y\to a$ or $Y\to YY$. We describe CM algorithms (including QDB) in terms of this normal form. CMs define a specific way that nodes in the guide tree are expanded into states, and how those states are connected within each node and to states in the next node in the guide tree. For example, a MATP node that deals with a consensus base pair contains six states called MATP_MP (a P state for matching the base pair), MATP_ML and MATP_MR (a L and R state for matching only the leftmost or rightmost base and deleting the right or left one, respectively), MATP_D (a D state for deleting the base pair), and MATP_IL and MATP_IR (L and R states with self-transitions, for inserting one or more residues to the left and/or right before going to the next node).

Thus a CM is a generative probabilistic model of homologous RNAs. A sequence is emitted starting at the root, moving downwards from state to state according to state transition probabilities, emitting residues and residue pairs according to emission probabilities, and bifurcating into substructures at bifurcation states. An important property of a CM is the states can be numbered from 0..*M*-1 (from root to leaves) such that for any state *v*, the states *y* that it can transit to must have indices *y*≥*v*. There are no cycles in a CM, other than self-transitions on insert states. This is the property that enables the recursive calculations that both CM DP alignment algorithms and QDB rely on.

Without any change in the above description, CMs apply to either global or local alignment, and to either pairwise alignment to single RNA queries or profile alignment to a consensus query structure of a multiple RNA sequence alignment. CMs for single RNA queries are derived identically to profiles of a consensus structure, differing only in the parameterization method [27]. Local structural alignment to substructures and truncated structures (as opposed to requiring a global alignment to the whole RNA structural model) is achieved by adding state transitions from the ROOT that permit entering the model at any internal consensus state with some probability, and state transitions from any internal consensus state to an END with some probability [26, 27].

### QDB algorithm

Observe that for any state *v*, we could enumerate all possible paths down the model from *v* to the END(s). Each path has a certain probability (the product of the transition probabilities used by the path), and it will emit a certain number *n* of residues (2 per P state, 1 per L or R state in the path). The sum of these path probabilities for each *n* defines a probability distribution $\gamma_{v}(n)$, the probability that the CM subgraph rooted at *v* will generate a subsequence of length *n*. Given a finite limit *Z* on maximum subsequence length (defined later), we can calculate $\gamma_{v}(n)$ by an efficient recursive algorithm, working from the leaves of the CM towards the root, and from smallest subsequences to largest:

| for *v*=*M*-1 down to 0: | | |
| --- | --- | --- |
| *v*= end state (*E*): |  |  |
|  |  | for *d*=1 to *Z* |
|  |  |  |
| *v*= bifurcation (*B*): | $\gamma_{v}(d)=\sum_{n=0}^{d} \gamma_{y}(n)*\gamma_{z}(d-n)$ | for *d*=0 to *Z* |
|  |  |  |
| else (*v*=*S*,*P*,*L*,*R*): | $\gamma_{v}(d)=0$ | for *d*=0 to |
|  | $\gamma_{v}(d)=\sum_{y\in Cv} \gamma_{y}(d-(\Delta_{v}^{L}+\Delta_{v}^{R}))*t_{v}(y)$ | for $d=(\Delta_{v}^{L}+\Delta_{v}^{R})$ to *Z* |

For example, if we are calculating $\gamma_{v}(d)$ where *v* is is a pair state, we know that *v* must emit a pair of residues then transit to a new state *y* (one of its possible transitions $C_{v}$), and then a subgraph rooted at *y* will have to account for the rest of the subsequence of length *d*-2. Therefore, $\gamma_{v}(d)$ must be the sum, over all possible states *y* in , of the transition probability $t_{v}(y)$ times the probability that the subtree rooted at *y* generates a subsequence of length *d*-2 – which is $\gamma_{y}(d-2)$, guaranteed to have already been calculated by the recursion. For the *B* state (bifurcation) calculation, indices *y* and *z* indicate the left and right S (start) state that bifurcation state *v* must connect to.

A band dmin(*v*)..dmax(*v*) of subsequence lengths that will be allowed for each state *v* is then defined as follows. A parameter β defines the threshold for the negligible probability mass that we are willing to allow outside the band. (The default value of β is set to , as described later.) We define dmin(*v*) and dmax(*v*) such that the cumulative left and right tails of $\gamma_{v}(n)$ contain less than a probability :

$$\int_{d=0}^{dmin(v)-1} \gamma_{v}(d)<\frac{\beta}{2},$$

$$\int_{d=dmax(v)+1}^{Z} \gamma_{v}(d)<\frac{\beta}{2}.$$

Larger values of β produce tighter bands and faster alignments, but at a cost of increased risk of missing the optimal alignment. β is the only free parameter that must be specified to QDB.

Because CMs have emitting self-loops (i.e. insert states), there is no finite limit on subsequence lengths. However, we must impose a finite limit *Z* to obtain a finite calculation. *Z* can be chosen to be sufficiently large that it does not affect dmax(*v*) for any state *v*. On a digital computer with floating point precision $\varepsilon$ (the largest value for which 1+$\varepsilon$ = 1), it suffices to guarantee that, for all *v*:

$$\frac{\sum_{d=Z+1}^{\infty} \gamma_{v}(d)}{\int_{d'=dmax(v)+1}^{Z} \gamma_{v}(d')}\leq\varepsilon$$

Empirically, we observe that the tails of the $\gamma_{v}(d)$ densities decrease approximately geometrically. We can estimate the mass remaining in the unseen tail by fitting a geometric tail to the observed density $\gamma_{v}(d)$. Our implementation starts with a reasonable guess at *Z* and verifies that the above condition is true for each *v*, assuming these geometrically decreasing tails; if it is not, *Z* is increased and bands are recalculated until it is.

A QDB calculation only needs to be performed once per query CM to set the bands. Overall, a QDB calculation requires Θ(*MZ*) in time and space; or equivalently, because both *M* and *Z* scale roughly linearly with the length *L* in residues of the query RNA, $\Theta(L^{2})$. The time and space requirement is negligible compared to the requirements of a typical CM search.

### Banded CYK database search algorithm for CMs

A standard algorithm for obtaining the maximum likelihood alignment (parse tree) of an SCFG to a target sequence is the Cocke-Younger-Kasami (CYK) dynamic programming algorithm [28, 29, 30]. Formally, CYK applies to SCFGs reduced to Chomsky normal form, and it aligns to the complete sequence. The CM database search algorithm is a CYK variant, specialized for the “normal form” of our seven types of RNA production rules, and for scanning long genomic sequences for high-scoring subsequences (hits) [14].

The CM search algorithm recursively calculates $\alpha_{v}(j,d)$, the log probability of the most likely CM parse subtree rooted at state *v* that generates (aligns to) the length *d* subsequence $x_{j-d+1}..x_{j}$ that ends at position *j* of target sequence *x* [15, 14]. This calculation initializes at the smallest subgraphs (*E* states) and shortest subsequences (*d*=0) and iterates upwards and outwards to progressively larger subtrees and longer subsequences up to a preset window size *W*. The outermost loop iterates over the end position *j* on the target sequence, enabling an efficient scan across a long target like a chromosome sequence. Banding is achieved simply by limiting all loops over possible subsequence lengths *d* to the bounds dmin(*v*)..dmax(*v*) derived in the band calculation algorithm, rather than all possible lengths 0..*W*. The banded version of the algorithm is as follows:

| Initialization (impose bands): for *j*=0 to *L*, *v*=*M*-1 down to 0: | |
| --- | --- |
| for *d*=0 to min((dmin(*v*)-1),*j*) | $\alpha_{v}(j,d)=-\infty;$ |
| for *d*=(dmax(*v*)+1) down to *j* | $\alpha_{v}(j,d)=-\infty.$ |
|  |  |
| Initialization at *d*=0: for *j*=0 to *L*, *v*=*M*-1 down to 0: | |
| *v*= end state (*E*): | $\alpha_{v}(j,0)=0;$ |
| *v*= bifurcation (*B*): | $\alpha_{v}(j,0)=\alpha_{y}(j,0)+\alpha_{z}(j,0);$ |
| *v*= delete or start (*D*,*S*): | $\alpha_{v}(j,0)=\max_{y\in C_{v}}[\alpha_{y}(j,0)+\log t_{v}(y)];$ |
| else (*v*=*P*,*L*,*R*): | $\alpha_{v}(j,0)=-\infty.$ |
|  |  |
| Recursion: for *j*=1 to *L*,*d*=max(1,dmin(*v*)) to min(dmax(*v*),*j*),*v*=*M*-1 down to 0 | |
| *v*= end state (*E*): | $\alpha_{v}(j,d)=-\infty;$ |
| *v*= bifurcation (*B*): | kmin=max(dmin(*z*),(*d*-dmax(*y*))), |
|  | kmax=min(dmax(*z*),(*d*-dmin(*y*))), |
|  | $\alpha_{v}(j,d)=\max_{k\min\leq k\leq k\max}[\alpha_{y}(j-k,d-k)+\alpha_{z}(j,k)];$ |
| *v*= delete or start (*D*,*S*): | $\alpha_{v}(j,d)=\max_{y\in C_{v}}[\alpha_{y}(j,d)+\log t_{v}(y)];$ |
| else (*v*=*P*,*L*,*R*): | $\alpha_{v}(j,d)=\max_{y\in C_{v}}[\alpha_{y}(j-\Delta_{v}^{R},d-(\Delta_{v}^{L}+\Delta_{v}^{R}))+\log t_{v}(y)]$ |
|  | $+\log e_{v}(x_{i},x_{j})$. |

For example, if we are calculating $\alpha_{v}(j,d)$ and *v* is a pair state (*P*), *v* will generate the basepair and transit to a new state *y* (one of its possible transitions ) which then will have to account for the smaller subsequence . The log odds score for a particular choice of next state *y* is the sum of three terms: an emission term $\log e_{v}(x_{j-d+1},x_{j})$, a transition term $\log t_{v}(y)$, and an already calculated solution for the smaller optimal parse tree rooted at *y*, $\alpha_{y}(j-1,d-2)$. The value assigned to $\alpha_{v}(j,d)$ is the maximum over all possible choices of child states *y* that *v* can transit to.

The *W* parameter defines the maximum size of a potential hit to a model. Previous infernal implementations required an *ad hoc* guess at a reasonable *W*. The band calculation algorithm delivers a probabilistically derived *W* for database search in dmax(0), the upper bound on the length of the entire sequence (the sequence generated from the root state of the CM).

QDB does not reduce the asymptotic computational complexity of the CM search algorithm. Both the banded algorithm and the original algorithm are $O(MW+BW^{2})$ memory and $O(L(MW+BW^{2}))$ time, for a model of *M* states containing *B* bifurcation states, window size *W* of residues, and target database length *L*. *M*, *B*, and *W* all scale with the query RNA length *N*, so roughly speaking, worst-case asymptotic time complexity is $O(LN^{3})$.

### Informative Dirichlet priors

The subsequence length distributions calculated by QDB depend on the CM’s transition probabilities. Transition probability parameter estimation is therefore crucial for obtaining predicted subsequence length bands that reflect real subsequence lengths in homologous RNA targets. Transition parameters in infernal are mean posterior estimates, combining (*ad hoc* weighted) observed counts from an input RNA alignment with a Dirichlet prior [26]. Previous to this work, infernal used an uninformative uniform Dirichlet transition prior, equivalent to the use of Laplace “plus-1” pseudo-counts. However, we found that transition parameters derived under a uniform prior inaccurately predict target subsequence lengths, as shown in an example in Figure 2. The problem is exacerbated when there are few sequences in the query alignment, when the choice of prior has more impact on mean posterior estimation. To alleviate this problem, we estimated informative single component Dirichlet prior densities for CM transition parameters, as follows.

The training data for transition priors consisted of the 381 seed alignments in the Rfam database, version 6.1 [17]. For each alignment, we built CM structures by infernal’s default procedure, and collected weighted counts of observed transitions in the implied parse trees of the training sequences. Considering all possible combinations of pairs of adjacent node types, there are 73 possible distinct types of transition probability distributions in CMs. To reduce this parameter space, we tied these 73 distributions into 36 groups by assuming that certain distributions were effectively equivalent. 36 Dirichlet densities were then estimated from these pooled counts by maximum likelihood as described in [31], with the exception that we optimize by conjugate gradient descent [32] rather than by expectation-maximization (EM). The results, including the Dirichlet parameters, are given in Table 1. Using these priors for transition probability parameter estimation results in an improvement in the utility of QDB calculations, often yielding tighter, yet accurate subsequence length distributions, as illustrated by anecdotal example in Figure 2.

We also estimated informative mixture Dirichlet density priors for emission probabilities. Emission probabilities have no effect on QDB, but informative emission priors should improve sensitivity and specificity of CM searches, as they do for profile hidden Markov models [33, 31]. We collected filtered counts of aligned single-stranded residues and base-pairs from annotated ribosomal RNA alignments from four alignments in the 2002 version of the European Ribosomal RNA Database [34, 35]: large subunit rRNA (LSU), bacterial/archaeal/plastid small subunit rRNA (SSU-bap), eukaryotic SSU rRNA (SSU-euk), and mitochondrial SSU rRNA (SSU-mito). These alignments were filtered, removing sequences in which either less than 40% of the base paired positions are present or more than 5% of the nucleotides are ambiguous, and removing selected sequences based on single-linkage-clustering such that no two sequences in a filtered alignment were greater than 80% identical (in order to remove closely related sequences). Summary statistics for the filtered alignments and collected counts in the training dataset are given in Table 2. These data were used to estimate a nine-component Dirichlet mixture prior for base pairs, and an eight-component Dirichlet mixture prior for single stranded residues. The base pair prior is given in Table 3, and the singlet residue prior is given in Table 4.

The reason to use two different datasets to estimate transition versus emission priors is the following. Rfam provides many different structural RNA alignments, but of uneven quality and varying depth (number of sequences). The European rRNA database provides a small number of different RNA alignments, but of high quality and great depth. A transition prior training set should be maximally diverse, so as not to bias any transition types toward any particular RNA structure, so we used the 381 different Rfam alignments for transitions. Emission prior estimation, in contrast, improves with alignment depth and accuracy, but does not require broad structural diversity per se, so we used rRNA data for emissions.

Inspection of the Dirichlet α parameters shows sensible trends. In the transition priors, transitions between main (consensus) states are now favored (higher α values) relative to insertions and deletions. In the base pair emission mixture prior, all components favor Watson-Crick and G-U pairs, with different components preferring different proportions of pairs in a particular covarying aligned column (for instance, component 1 likes all four Watson-Crick pairs, component 2 describes covarying conservation of CG,UA,UG pairs, and component 3 specifically likes conserved CG pairs), and the mean α parameters prefer GC/CG pairs over AU/UA pairs. In the singlet emission mixture prior, some components are capturing strongly conserved residues (component 1 favors conserved U’s, for example) while other components favor more variation (components 4 and 5, for example), and the marginal α parameters show a strong A bias, reflecting the known bias for adenine in single-stranded positions of structural RNAs (especially ribosomal RNAs).

There is redundancy between some components (notably 5 and 8 in the base pair mixture and 2, 3 and 8 in the singlet mixture). This is typical for statistical mixture estimation, which (unlike, say, principal components analysis) does not guarantee independence between components. The decision to use nine pair and eight singlet components was empirical, as these priors performed better than priors with fewer components on the benchmark we describe below (data not shown).

Note that all singlet positions are modeled with one singlet mixture prior distribution, and all base pairs are modeled with one base pair mixture prior. These priors do not distinguish between singlet residues in different types of loops, for example, nor between a stem-closing base pair versus other base pairs. In the future it may prove advantageous to adopt more complex priors to capture effects of structural context on base pair and singlet residue preference.

In another step to increase sensitivity and specificity of the program, we adopted the “entropy weighting” technique described for profile HMMs [36] for estimating the total effective sequence number for an input query alignment. This is an *ad hoc* method for reducing the information content per position of a model, which helps a model that has been trained on closely related sequences to recognize distantly related homologues [37]. In entropy weighting, one reduces the total effective sequence number (which would normally be the actual number of sequences in the input alignment), thereby increasing the influence of the Dirichlet priors, flattening the transition and emission distributions, and reducing the overall information content. We approximate a model’s entropy as the mean entropy per consensus residue, as follows. Let *C* be the set of all MATP_MP states emitting consensus base pairs (*a*,*b*), and let *D* be the set of all MATL_ML and MATR_MR states emitting consensus singlets (*a*); the entropy is then calculated as:

$$\frac{-\sum_{v\in C} \int_{a,b} e_{v}(a,b)\log e_{v}(a,b)-\sum_{v\in D} \int_{a} e_{v}(a)\log e_{v}(a)}{2|C|+|D|}$$

For each input multiple alignment, the effective sequence number is set (by bracketing and binary search) so as to obtain a specified target entropy. The target entropy for infernal is a free parameter, which we optimized on the benchmark described below to identify our default value of 1.46 bits.

### Benchmarking

To assess the effect of QDB, informative priors, and entropy weighting on the speed, sensitivity, and specificity of RNA similarity searches, we designed a benchmark based on the Rfam database [17]. The benchmark was designed so that we would test many RNA query/target pairs, with each query consisting of a given RNA sequence alignment, and each target consisting of a distantly related RNA homolog buried in a context of a random genome-like background sequence.

We started with seed alignments from Rfam version 7.0. In each alignment, sequences shorter than 70% the median length were removed. We clustered the sequences in each family by single-linkage-clustering by % identity (as calculated from the given Rfam alignment), then split the clusters such that the training set and test sequences satisfied three conditions: 1) no training/test sequence pair is more than 60% identical; 2) no test sequence pair is greater than 70% identical; 3) at least 5 sequences are in the training set. Fifty-one families satisfy these criteria (listed in Table 5), giving us 51 different query alignments (containing 5 to 1080 sequences each) and 450 total test sequences (from 1 to 66 per query). We embedded the test sequences in a one megabase “pseudo-genome” consisting of twenty 50 kilobase “chromosomes”, generated as independent, identically distributed (iid) random sequences with uniform base frequencies. The 450 test sequences were embedded into this sequence by replacement, by randomly choosing a chromosome, orientation, and start position, and disallowing overlaps between test sequences. The total length of the 450 test sequences is 101,855 nt, leaving 898,145 nt of random background sequence.

The benchmark proceeds by first building a CM for each query alignment, then searching the pseudo-genome with each CM in local alignment mode. All hits above a threshold of 8.0 in raw bit score for each of the 51 queries were sorted by score into 51 ranked family specific lists, as well as one ranked master list of all 51 sets of scores. Each hit is classified into one of three categories, “positive”, “ignore”, or “negative”. A “positive” is a hit that significantly overlaps with a true test sequence from the same family as the query. An “ignore” is a hit that significantly overlaps with a test sequence from a different family, where “significantly overlap” means that the length of overlap between two sequences (either two hits, or one hit and one test sequence embedded in the pseudo-genome) is more than 50% the length of the shorter sequence. (Although it would be desirable to measure the false positive rate on nonhomologous structural RNAs, we cannot be sure that any given pair of Rfam families is truly nonhomologous. Like most sequence family databases, Rfam is clustered computationally, and more sensitive methods will reveal previously unsuspected relationships that should not be benchmarked as “false positives”.) A “negative” is a hit that is not a positive or an ignore. For any two negatives that significantly overlap, only the one with the better score is counted.

The minimum error rate (MER) (“equivalence score”) [38] was used as a measure of benchmark performance. The MER score is defined as the minimum sum of the false positives (negative hits above the threshold) and false negatives (true test sequences which have no positive hit above the threshold), at all possible choices of score threshold. The MER score is a combined measure of sensitivity and specificity, where a lower MER score is better. We calculate two kinds of MER scores. For a *family-specific* MER score, we choose a different optimal threshold in each of the 51 ranked lists, and for a *summary* MER score, we choose a single optimal threshold in the master list of all hits. The summary MER score is the more relevant measure of our current performance, because it demands a single query-independent bit score threshold for significance. A family-specific MER score reflects the performance that could be achieved if infernal provided P-values (currently it reports only raw bit scores).

For comparison, blastn was also benchmarked on these data using a family-pairwise-search (FPS) procedure [39]. For each query alignment, each training sequence is used as a query sequence to search the pseudo-genome, all hits with an E-value of less than 1.0 were sorted by increasing E-value, and the lowest E-value positive hit to a given test sequence is counted.

Using this benchmark, we addressed several questions about QDB’s performance.

What is the best setting of the single QDB free parameter, β, which specifies how much probability mass to sacrifice? Figure 3 shows the average speedup per family and summary MER score as a function of varying β. There is no clear choice. The choice of β is a tradeoff of accuracy for speed. We chose a default of as a reasonable value that obtains a modest speedup with minimal loss of accuracy.

How well does QDB accelerate CM searches? Figure 4 shows the time required for searching the 1 Mb benchmark target sequence with each of the 51 models, as a function of the average query RNA length. QDB reduces the average-case running time complexity of the CM search algorithm from to . Observed accelerations relative to the standard algorithm range from 1.4-fold (for the IRE, iron response element) to 12.7-fold (for the 5’ domain of SSU rRNA), with an average speed-up per family of 4.2-fold. In total search time for the benchmark (sum of all 51 searches), the acceleration is six-fold, because large queries have disproportionate effect on the total time.

How much does QDB impact sensitivity and specificity? Optimal alignments are not guaranteed to lie within QDB’s high-probability bands. This is expected to compromise sensitivity. The hope is that QDB’s bands are sufficiently wide and accurate that the loss is negligible. Figure 5 shows ROC plots (sensitivity versus false positive rate) on the benchmark for the new version of infernal (version 0.72) in standard versus QDB mode. These plots are nearly superposed, showing that the loss in accuracy is small at the default QDB setting of .

How much do our changes in parameterization (the addition of informative Dirichlet priors and entropy weighting) improve sensitivity and specificity? Figure 5 shows that the new infernal 0.72 is a large improvement over the previous infernal version 0.55, independent of QDB. (On average, in this benchmark, infernal 0.55 is no better than a family-pairwise-search with blastn.) Table 6 breaks this result down in more detail, showing summary and family-specific MER scores for a variety of combinations of prior, entropy weighting, and QDB. These results show that both informative priors and entropy weighting individually contributed large improvements in sensitivity and specificity.

## Discussion

CM searches take a long time, and this is the most limiting factor in using the infernal software to identify RNA similarities. Prior to this work, infernal 0.55 required 508 CPU-hours to search 51 models against just 1 megabase of sequence in our benchmarks (Table 5). Using QDB with β banding cutoffs that do not appreciably compromise sensitivity and specificity, infernal 0.72 offers a six-fold speedup, performing the benchmark in 85 hours. Our eventual goal is to enable routine genome annotation of structural RNAs: to be able to search thousands of RNA models against complete genome sequences. A search of all 503 Rfam 7.0 models against the 3 GB human genome with infernal 0.72 in QDB mode would take on the order of 300 CPU-years (down from 1800 with infernal 0.55). We need to be able to do it in at most a few days, so we still need to increase CM search speed by five orders of magnitude. Thus, the QDB algorithm is a partial but certainly not complete solution to the problem. However, QDB combines synergistically with other acceleration techniques. Parallelization, on large clusters (though prohibitively expensive for all but a few centers) could give us further acceleration of three orders of magnitude. Software improvement (code optimization) will also contribute, but probably only about two-fold. Hardware improvements will contribute about two-fold per year or so so long as Moore’s law continues. Finally, QDB is complementary to the filtering methods recently described by Weinberg and Ruzzo [20, 21, 22]. We view QDB as part of a growing suite of approaches that we can combine to accelerate infernal.

Is it really worth burning all this CPU time in the first place? Do CM searches identify structural RNA homologies that other methods miss? Obviously we think so, but one would like to see convincing results. For large, diverse RNA families like tRNA, where a CM can be trained on over a thousand well-aligned sequences with a well-conserved consensus secondary structure, CM approaches have been quite powerful. The state of the art in large scale tRNA gene identification remains the CM-based program trnascan-se [1], and CMs were also used, for example, to discover the divergent tRNA for pyrrolysine, the “22nd amino acid” [40]. But Figure 5 shows that on average, over 51 more or less “typical” RNA families of various sizes and alignment quality, infernal 0.55 was actually no better than doing a family-pairwise-search with blastn. Until recently, we have spent relatively little effort on how infernal parameterizes its models, and relatively more on reducing its computational requirements [16], so previous versions of infernal have performed best where naive parameterization works best: on very large, high-quality alignments of hundreds of sequences, which are atypical of many interesting homology search problems.

In this work, partly because the level of acceleration achieved by QDB is sensitive to transition parameterization, we have brought infernal parameterization close to the state of the art in profile HMMs, by introducing mixture Dirichlet priors [31] and entropy weighting [36]. This resulted in a large improvement in the sensitivity and specificity of searches, as judged by our benchmark (Figure 5). The difference between infernal and family-pairwise blastn now appears pronounced for average-case behavior, not just best-case behavior. However, while we trust our benchmarking to tell us we have greatly improved infernal relative to previous versions of itself, our benchmarking does not allow us to draw firm conclusions about our performance relative to other software. For that, we prefer to see independent benchmarks. Benchmarks by tool developers are notoriously biased, and however honest we may try to be, some biases are essentially unavoidable. For one thing, establishing an internal benchmark for ongoing code development creates an insidious form of training on the test set, because we accept code changes that improve benchmark performance. In particular, we set the entropy weighting target of 1.46 bits and the numbers of mixture prior components by optimizing against our benchmark. Further, our benchmark does not use a realistic model for the background sequence of the “pseudo-genome”, because we construct the background as a homogeneous iid (independent, identically distributed) sequence, and this poorly reflects the heterogeneous and repetitive nature of genomic sequence. This benchmark should be sufficient for an internal comparison of versions 0.55 and 0.72 of infernal, because we have not altered how infernal deals with heterogeneous compositional bias. But we cannot safely draw conclusions from our simple benchmark about the relative performance of infernal and blast on real searches, for example, because blast may (and in fact does) treat sequence heterogeneity better than infernal does. In this regard, currently we are aware of only one independent benchmark BRaliBase III [41]. BRaliBase III consists of many different query alignments of 5 or 20 RNA sequences, drawn from three different RNA families (U5, 5S rRNA, and tRNA). These authors’ results broadly confirm our internal observations: while infernal 0.55 showed mediocre performance compared to blastn and several other tools, a recent version of infernal stood out as a superior method for RNA similarity search.

Nonetheless, though infernal 0.72 shows large improvements in speed, sensitivity, and specificity over previous versions, there are numerous areas where we need to improve further.

A significant gap in our current implementation is that infernal reports only raw bit scores, and does not yet report expectation values (E-values). CM local alignment scores empirically follow a Gumbel (extreme value) distribution [27], just as local sequence alignment scores do [42], so there are no technical hurdles in implementing E-values. This will be an immediate focus for the next version of infernal. E-value calculations not only have the effect of reporting statistical significance (more meaningful to a user than a raw bit score), but they also normalize each family’s score distribution into a more consistent overall rank order, because different query models exhibit different null distributions (particularly in the location parameter of the Gumbel distribution). We therefore expect E-values to contribute a large increase in performance whenever a single family-independent threshold is set. Table 6 roughly illustrates the expected gain, by showing the large difference between summary MER scores and family-specific MER scores.

Parameterization of both CMs and profile HMMs remains problematic, because these methods continue to assume that training sequences are statistically independent, when in fact they are related (often strongly so) by phylogeny. Methods like sequence weighting and entropy weighting do help, but they are *ad hoc* hacks: unsatisfying and unlikely to be optimal. Even mixture Dirichlet priors, though they appear to be mathematically sophisticated, fundamentally assume that observed counts are drawn as independent multinomial samples, and therefore the use of Dirichlet priors is fundamentally flawed. Probabilistic phylogenetic inference methodology needs to be integrated with profile search methods. This is an area of active research [43, 44, 45] in which important challenges remain, particularly in the treatment of insertions and deletions.

Finally, QDB is not the only algorithmic acceleration method we can envision. Michael Brown described a complementary banding method to accelerate his SCFG-based rnacad ribosomal RNA alignment software [25], in which he uses profile HMM based sequence alignment to the target to determine bands where the more rigorous SCFG-based alignment should fall (because some regions of the alignment are well-determined based solely on sequence alignment). The gapped blast algorithm (seed word hits, ungapped hit extension, and banded dynamic programming) can conceivably be extended from two-dimensional sequence alignment to three-dimensional CM dynamic programming lattices. Developing such algorithms – and incorporating them into a widely useful, freely available codebase – are priorities for us.

## Materials and Methods

The version and options used for blast in our benchmark are WU-BLASTN-2.0MP –kap -W=7. For infernal, versions 0.55 and 0.72 were used as indicated. The complete infernal software package, including documentation and the Rfam-based benchmark described here, may be downloaded from http://infernal.janelia.org. It is developed on GNU/Linux operating systems but should be portable to any POSIX-compliant operating system, including Mac OS/X. It is freely licensed under the GNU General Public License.

The ANSI C code we used for estimating maximum likelihood mixture Dirichlet priors depends on a copyrighted and nonredistributable implementation of the conjugate gradient descent algorithm from Numerical Recipes in C [32]. Our code, less the Numerical Recipes routine, is freely available upon request.

## Acknowledgements

We thank Elena Rivas for critical comments on the manuscript.

## Funding

EPN gratefully acknowledges financial support from an NIH NHGRI Institutional Training Grant in Genomic Science, T32-HG000045. Work in SRE’s lab in Washington University in St. Louis, where this work was initiated, was supported by NIH NHGRI RO1-HG01363, HHMI, and Alvin Goldfarb.

**References**

[1]	Lowe TM, Eddy SR (1997) tRNAscan-SE: A program for improved detection of transfer RNA genes in genomic sequence. Nucl Acids Res 25:955–964.

[2]	Laslett D, Canback B (2004) ARAGORN, a program to detect tRNA genes and tmRNA genes in nucleotide sequences. Nucl Acids Res 32:11–16.

[3]	Lowe TM, Eddy SR (1999) A computational screen for methylation guide snoRNAs in yeast. Science 283:1168–1171.

[4]	Schattner P, Barberan-Soler S, Lowe TM (2006) A computational screen for mammalian pseudouridylation guide H/ACA RNAs. RNA 12:15–25.

[5]	Lai EC, Tomancak P, Williams RW, Rubin GM (2003) Computational identification of *Drosophila* microRNA genes. Genome Biol 4:R42.

[6]	Lim LP, Glasner ME, Yekta S, Burge CB, Bartel DP (2003) Vertebrate microRNA genes. Science 299:1540.

[7]	Regalia M, Rosenblad MA, Samuelsson T (2002) Prediction of signal recognition particle RNA genes. Nucl Acids Res 30:3368–3377.

[8]	Ermolaeva MD, Khalak HG, White O, Smith HO, Salzberg SL (2000) Prediction of transcription terminators in bacterial genomes. J Mol Biol 301:27–33.

[9]	Macke TJ, Ecker DJ, Gutell RR, Gautheret D, Case DA, et al. (2001) RNAMotif, an RNA secondary structure definition and search algorithm. NAR 29:4724–4735.

[10]	Gautheret D, Lambert A (2001) Direct RNA motif definition and identification from multiple sequence alignments using secondary structure profiles. J Mol Biol 313:1003–1011.

[11]	Zhang S, Haas B, Eskin E, Bafna V (2005) Searching genomes for noncoding RNA using FastR. IEEE/ACM Trans Comput Biol Bioinform 2:366–379.

[12]	Altschul SF, Madden TL, Schaffer AA, Zhang J, Zhang Z, et al. (1997) Gapped BLAST and PSI-BLAST: A new generation of protein database search programs. Nucl Acids Res 25:3389–3402.

[13]	Sakakibara Y, Brown M, Hughey R, Mian IS, Sjolander K, et al. (1994) Stochastic context-free grammars for tRNA modeling. Nucl Acids Res 22:5112–5120.

[14]	Durbin R, Eddy SR, Krogh A, Mitchison GJ (1998) Biological Sequence Analysis: Probabilistic Models of Proteins and Nucleic Acids. Cambridge UK: Cambridge University Press.

[15]	Eddy SR, Durbin R (1994) RNA sequence analysis using covariance models. Nucl Acids Res 22:2079–2088.

[16]	Eddy SR (2002) A memory-efficient dynamic programming algorithm for optimal alignment of a sequence to an RNA secondary structure. BMC Bioinformatics 3:18.

[17]	Griffiths-Jones S, Moxon S, Marshall M, Khanna A, Eddy SR, et al. (2005) Rfam: Annotating non-coding RNAs in complete genomes. Nucl Acids Res 33:D121–D141.

[18]	Sonnhammer ELL, Eddy SR, Birney E, Bateman A, Durbin R (1998) Pfam: Multiple sequence alignments and HMM-profiles of protein domains. Nucl Acids Res 26:320–322.

[19]	Finn RD, Mistry J, Schuster-Bockler B, Griffiths-Jones S, Hollich V, et al. (2006) Pfam: Clans, web tools and services. Nucl Acids Res 34:D247–D251.

[20]	Weinberg Z, Ruzzo WL (2004) Exploiting conserved structure for faster annotation of non-coding RNAs without loss of accuracy. Bioinformatics 20 Suppl. 1:I334–I341.

[21]	Weinberg Z, Ruzzo WL (2004) Faster genome annotation of non-coding RNA families without loss of accuracy. RECOMB ’04 :243–251.

[22]	Weinberg Z, Ruzzo WL (2006) Sequence-based heuristics for faster annotation of non-coding RNA families. Bioinformatics 22:35–39.

[23]	Zhang S, Borovok I, Aharonowitz Y, Sharan R, Bafna V (2006) A sequence-based filtering method for ncRNA identification and its application to searching for riboswitch elements. Bioinformatics 22:e557–e565.

[24]	Brudno M, Do CB, Cooper GM, Kim MF, Green E, NISC Comparative Sequencing Program, Sidow A, Batzoglou S (2003) LAGAN and multi-LAGAN: efficient tools for large-scale multiple alignment of genomic DNA. Genome Res 13:721–731.

[25]	Brown MP (2000) Small subunit ribosomal RNA modeling using stochastic context-free grammars. Proc Int Conf on Intelligent Systems in Molecular Biology 8:57–66.

[26]	Eddy SR (2003). The Infernal user’s guide. [http://infernal.janelia.org/].

[27]	Klein RJ, Eddy SR (2003) RSEARCH: finding homologs of single structured RNA sequences. BMC Bioinformatics 4:44.

[28]	Kasami T (1965) An efficient recognition and syntax algorithm for context-free algorithms. Technical Report AFCRL-65-758, Air Force Cambridge Research Lab, Bedford, Mass.

[29]	Younger DH (1967) Recognition and parsing of context-free languages in time . Information and Control 10:189–208.

[30]	Hopcroft JE, Ullman JD (1979) Introduction to Automata Theory, Languages, and Computation. Addison-Wesley.

[31]	Sjölander K, Karplus K, Brown M, Hughey R, Krogh A, et al. (1996) Dirichlet mixtures: A method for improving detection of weak but significant protein sequence homology. Comput Applic Biosci 12:327–345.

[32]	Press WH, Teukolsky SA, Vetterling WT, Flannery BP (1993) Numerical Recipes in C: The Art of Scientific Computing. Cambridge University Press.

[33]	Brown M, Hughey R, Krogh A, Mian IS, Sjolander K, et al. (1993) Using dirichlet mixture priors to derive hidden Markov models for protein families. In: Hunter L, Searls D, Shavlik J, editors, Proceedings of the First International Conference on Intelligent Systems for Molecular Biology. Menlo Park, CA: AAAI, pp. 47–55.

[34]	Wuyts J, Rijk PD, de Peer YV, Winkelmans T, Wachter RD (2001) The European large subunit ribosomal RNA database. Nucl Acids Res 29:175–177.

[35]	Wuyts J, de Peer YV, Winkelmans T, Wachter RD (2002) The European database on small subunit ribosomal RNA. Nucl Acids Res 30:183–185.

[36]	Karplus K, Barrett C, Hughey R (1998) Hidden Markov models for detecting remote protein homologies. Bioinformatics 14:846–856.

[37]	Altschul SF (1991) Amino acid substitution matrices from an information theoretic perspective. J Mol Biol 219:555–565.

[38]	Pearson WR (1995) Comparison of methods for searching protein sequence databases. Protein Sci 4:1145–1160.

[39]	Grundy WN (1998) Homology detection via family pairwise search. J Comput Biol 5:479–491.

[40]	Srinivasan G, James CM, Krzycki JA (2002) Pyrrolysine encoded by UAG in archaea: Charging of a UAG-decoding specialized tRNA. Science 296:1459–1462.

[41]	Freyhult EK, Bollback JP, Gardner PP (2007) Exploring genomic dark matter: A critical assessment of the performance of homology search methods on noncoding RNA. Genome Res 17:117–125.

[42]	Karlin S, Altschul SF (1993) Applications and statistics for multiple high-scoring segments in molecular sequences. Proc Natl Acad Sci USA 90:5873–5877.

[43]	Holmes I (2004) A probabilistic model for the evolution of RNA structure. BMC Bioinformatics 5:166.

[44]	Holmes I (2005) Using evolutionary expectation maximization to estimate indel rates. Bioinformatics 21:2294–2300.

[45]	Rivas E (2005) Evolutionary models for insertions and deletions in a probabilistic modeling framework. BMC Bioinformatics 6:63.

|  | tied | dist | grp | node | next |  | Dirichlet α parameters | | | | | | | | | | | | | |
| --- | --- | --- | --- | --- | --- | --- | --- | --- | --- | --- | --- | --- | --- | --- | --- | --- | --- | --- | --- | --- |
| # | to | cts | cts | state | node |  | \|α\| | *t* | |  | *t* |  | *t* |  | *t* |  | *t* |  | *t* |  |
| 1 |  | 6 | 11 | MATP_MP | BIF |  | 0.5509 | | IL | 0.1229 | IR | 0.0001 | B | 0.8770 |  |  |  |  |  |  |
| 2 |  | 7023 | 7119 | MATP_MP | MATP |  | 7.2986 | | IL | 0.0023 | IR | 0.0024 | MP | 0.9816 | ML | 0.0056 | MR | 0.0046 | D | 0.0035 |
| 3 |  | 1600 | 1830 | MATP_MP | MATL |  | 1.5914 | | IL | 0.0179 | IR | 0.0155 | ML | 0.9200 | D | 0.0466 |  |  |  |  |
| 4 |  | 145 | 195 | MATP_MP | MATR |  | 1.9038 | | IL | 0.0173 | IR | 0.0073 | MR | 0.8903 | D | 0.0852 |  |  |  |  |
| 5 | 1 | 2 | 11 | MATP_MP | END |  | 0.5509 | | IL | 0.1229 | IR | 0.0001 | E | 0.8770 |  |  |  |  |  |  |
| 6* |  | 1 | 2 | MATP_ML | BIF |  | 3.0000 | | IL | 0.3333 | IR | 0.3333 | B | 0.3333 |  |  |  |  |  |  |
| 7 |  | 577 | 577 | MATP_ML | MATP |  | 0.6941 | | IL | 0.0131 | IR | 0.0103 | MP | 0.4032 | ML | 0.4983 | MR | 0.0115 | D | 0.0636 |
| 8 |  | 133 | 133 | MATP_ML | MATL |  | 0.9316 | | IL | 0.0739 | IR | 0.0651 | ML | 0.7038 | D | 0.1571 |  |  |  |  |
| 9 |  | 15 | 15 | MATP_ML | MATR |  | 0.3272 | | IL | 0.1884 | IR | 0.0432 | MR | 0.4082 | D | 0.3602 |  |  |  |  |
| 10* | 6 | 1 | 2 | MATP_ML | END |  | 3.0000 | | IL | 0.3333 | IR | 0.3333 | E | 0.3333 |  |  |  |  |  |  |
| 11* |  | 1 | 2 | MATP_MR | BIF |  | 3.0000 | | IL | 0.3333 | IR | 0.3333 | B | 0.3333 |  |  |  |  |  |  |
| 12 |  | 531 | 531 | MATP_MR | MATP |  | 0.7987 | | IL | 0.0079 | IR | 0.0190 | MP | 0.3241 | ML | 0.0193 | MR | 0.5631 | D | 0.0666 |
| 13 |  | 151 | 151 | MATP_MR | MATL |  | 0.6933 | | IL | 0.0357 | IR | 0.0699 | ML | 0.3066 | D | 0.5879 |  |  |  |  |
| 14 |  | 15 | 15 | MATP_MR | MATR |  | 0.3574 | | IL | 0.0582 | IR | 0.0002 | MR | 0.7629 | D | 0.1787 |  |  |  |  |
| 15* | 11 | 1 | 2 | MATP_MR | END |  | 3.0000 | | IL | 0.3333 | IR | 0.3333 | E | 0.3333 |  |  |  |  |  |  |
| 16* |  | 0 | 2 | MATP_D | BIF |  | 3.0000 | | IL | 0.3333 | IR | 0.3333 | B | 0.3333 |  |  |  |  |  |  |
| 17 |  | 575 | 575 | MATP_D | MATP |  | 0.5450 | | IL | 0.0019 | IR | 0.0047 | MP | 0.0857 | ML | 0.0534 | MR | 0.0528 | D | 0.8015 |
| 18 |  | 149 | 149 | MATP_D | MATL |  | 0.5831 | | IL | 0.0421 | IR | 0.0526 | ML | 0.2080 | D | 0.6973 |  |  |  |  |
| 19 |  | 14 | 14 | MATP_D | MATR |  | 0.1164 | | IL | 0.0001 | IR | 0.0001 | MR | 0.2439 | D | 0.7559 |  |  |  |  |
| 20* | 16 | 2 | 2 | MATP_D | END |  | 3.0000 | | IL | 0.3333 | IR | 0.3333 | E | 0.3333 |  |  |  |  |  |  |
| 21 |  | 2 | 4 | MATP_IL | BIF |  | 1.4397 | | IL | 0.6553 | IR | 0.0445 | B | 0.3002 |  |  |  |  |  |  |
| 22 |  | 121 | 126 | MATP_IL | MATP |  | 0.9402 | | IL | 0.1673 | IR | 0.1394 | MP | 0.5904 | ML | 0.0443 | MR | 0.0259 | D | 0.0327 |
| 23 |  | 114 | 119 | MATP_IL | MATL |  | 0.8046 | | IL | 0.3108 | IR | 0.1936 | ML | 0.4610 | D | 0.0346 |  |  |  |  |
| 24 |  | 14 | 15 | MATP_IL | MATR |  | 1.0926 | | IL | 0.1419 | IR | 0.0501 | MR | 0.6538 | D | 0.1541 |  |  |  |  |
| 25 | 21 | 2 | 4 | MATP_IL | END |  | 1.4397 | | IL | 0.6553 | IR | 0.0445 | E | 0.3002 |  |  |  |  |  |  |
| 26 |  | 1 | 31 | MATP_IR | BIF |  | 0.9361 | | IR | 0.2827 | B | 0.7173 |  |  |  |  |  |  |  |  |
| 27 |  | 145 | 227 | MATP_IR | MATP |  | 1.5494 | | IR | 0.1884 | MP | 0.7090 | ML | 0.0165 | MR | 0.0588 | D | 0.0273 |  |  |
| 28 |  | 129 | 701 | MATP_IR | MATL |  | 1.6332 | | IR | 0.3681 | ML | 0.5752 | D | 0.0566 |  |  |  |  |  |  |
| 29 |  | 8 | 160 | MATP_IR | MATR |  | 1.2428 | | IR | 0.2633 | MR | 0.6809 | D | 0.0558 |  |  |  |  |  |  |
| 30 | 26 | 0 | 31 | MATP_IR | END |  | 0.9361 | | IR | 0.2827 | E | 0.7173 |  |  |  |  |  |  |  |  |
| 31 |  | 108 | 1130 | MATL_ML | BIF |  | 1.2298 | | IL | 0.0078 | B | 0.9922 |  |  |  |  |  |  |  |  |
| 32 |  | 420 | 1319 | MATL_ML | MATP |  | 2.4162 | | IL | 0.0132 | MP | 0.9520 | ML | 0.0150 | MR | 0.0129 | D | 0.0070 |  |  |
| 33 |  | 19013 | 19371 | MATL_ML | MATL |  | 1.8632 | | IL | 0.0082 | ML | 0.9711 | D | 0.0207 |  |  |  |  |  |  |
| 34 |  | 859 | 6692 | MATL_ML | MATR |  | 72.1283 | | IL | 0.0058 | MR | 0.9755 | D | 0.0187 |  |  |  |  |  |  |
| 35 | 31 | 801 | 1130 | MATL_ML | END |  | 1.2298 | | IL | 0.0078 | E | 0.9922 |  |  |  |  |  |  |  |  |
| 36 |  | 28 | 172 | MATL_D | BIF |  | 6.8008 | | IL | 0.0029 | B | 0.9971 |  |  |  |  |  |  |  |  |
| 37 |  | 103 | 103 | MATL_D | MATP |  | 0.7288 | | IL | 0.0321 | MP | 0.5730 | ML | 0.0536 | MR | 0.1654 | D | 0.1758 |  |  |
| 38 |  | 3152 | 3152 | MATL_D | MATL |  | 0.4101 | | IL | 0.0138 | ML | 0.3105 | D | 0.6756 |  |  |  |  |  |  |
| 39 |  | 154 | 154 | MATL_D | MATR |  | 0.6736 | | IL | 0.0203 | MR | 0.6014 | D | 0.3782 |  |  |  |  |  |  |
| 40 | 36 | 144 | 172 | MATL_D | END |  | 6.8008 | | IL | 0.0029 | E | 0.9971 |  |  |  |  |  |  |  |  |
| 41 | 26 | 13 | 31 | MATL_IL | BIF |  | 0.9361 | | IL | 0.2827 | B | 0.7173 |  |  |  |  |  |  |  |  |
| 42 | 27 | 35 | 227 | MATL_IL | MATP |  | 1.5494 | | IL | 0.1884 | MP | 0.7090 | ML | 0.0588 | MR | 0.0165 | D | 0.0273 |  |  |
| 43 | 28 | 549 | 701 | MATL_IL | MATL |  | 1.6332 | | IL | 0.3681 | ML | 0.5752 | D | 0.0566 |  |  |  |  |  |  |
| 44 | 29 | 45 | 160 | MATL_IL | MATR |  | 1.2428 | | IL | 0.2633 | MR | 0.6809 | D | 0.0558 |  |  |  |  |  |  |
| 45 | 26 | 0 | 31 | MATL_IL | END |  | 0.9361 | | IL | 0.2827 | E | 0.7173 |  |  |  |  |  |  |  |  |
| 46 | 31 | 206 | 1130 | MATR_MR | BIF |  | 1.2298 | | IR | 0.0078 | B | 0.9922 |  |  |  |  |  |  |  |  |
| 47 | 32 | 848 | 1319 | MATR_MR | MATP |  | 2.4162 | | IR | 0.0132 | MP | 0.9520 | ML | 0.0150 | MR | 0.0129 | D | 0.0070 |  |  |
| 48 | 34 | 5833 | 6692 | MATR_MR | MATR |  | 2.1283 | | IR | 0.0058 | MR | 0.9755 | D | 0.0187 |  |  |  |  |  |  |
| 49 |  | 39 | 39 | MATR_D | BIF |  | 0.4664 | | IR | 0.0463 | B | 0.9537 |  |  |  |  |  |  |  |  |
| 50 |  | 176 | 176 | MATR_D | MATP |  | 0.8689 | | IR | 0.0245 | MP | 0.6126 | ML | 0.1269 | MR | 0.0471 | D | 0.1890 |  |  |
| 51 |  | 771 | 771 | MATR_D | MATR |  | 0.4869 | | IR | 0.0119 | MR | 0.3373 | D | 0.6507 |  |  |  |  |  |  |
| 52 | 26 | 15 | 31 | MATR_IR | BIF |  | 0.9361 | | IR | 0.2827 | B | 0.7173 |  |  |  |  |  |  |  |  |
| 53 | 27 | 39 | 227 | MATR_IR | MATP |  | 1.5494 | | IR | 0.1884 | MP | 0.7090 | ML | 0.0165 | MR | 0.0588 | D | 0.0273 |  |  |
| 54 | 29 | 107 | 160 | MATR_IR | MATR |  | 1.2428 | | IR | 0.2633 | MR | 0.6809 | D | 0.0558 |  |  |  |  |  |  |
| 55 |  | 338 | 338 | BEGL_S | MATP |  | 5.0422 | | MP | 0.9579 | ML | 0.0121 | MR | 0.0183 | D | 0.0117 |  |  |  |  |
| 56 | 31 | 15 | 1130 | BEGR_S | BIF |  | 1.2298 | | IL | 0.0078 | B | 0.9922 |  |  |  |  |  |  |  |  |
| 57 | 32 | 51 | 1319 | BEGR_S | MATP |  | 2.4162 | | IL | 0.0132 | MP | 0.9520 | ML | 0.0150 | MR | 0.0129 | D | 0.0070 |  |  |
| 58 | 33 | 358 | 19371 | BEGR_S | MATL |  | 1.8632 | | IL | 0.0082 | ML | 0.9711 | D | 0.0207 |  |  |  |  |  |  |
| 59 | 26 | 2 | 31 | BEGR_IL | BIF |  | 0.9361 | | IL | 0.2827 | B | 0.7173 |  |  |  |  |  |  |  |  |
| 60 | 27 | 3 | 227 | BEGR_IL | MATP |  | 1.5494 | | IL | 0.1884 | MP | 0.7090 | ML | 0.0588 | MR | 0.0165 | D | 0.0273 |  |  |
| 61 | 28 | 19 | 701 | BEGR_IL | MATL |  | 1.6332 | | IL | 0.3681 | ML | 0.5752 | D | 0.0566 |  |  |  |  |  |  |
| 62 | 1 | 3 | 11 | ROOT_S | BIF |  | 0.5509 | | IL | 0.1229 | IR | 0.0001 | B | 0.8770 |  |  |  |  |  |  |
| 63 | 2 | 96 | 7119 | ROOT_S | MATP |  | 7.2986 | | IL | 0.0023 | IR | 0.0024 | MP | 0.9816 | ML | 0.0056 | MR | 0.0046 | D | 0.0035 |
| 64 | 3 | 230 | 1830 | ROOT_S | MATL |  | 1.5914 | | IL | 0.0179 | IR | 0.0155 | ML | 0.9200 | D | 0.0466 |  |  |  |  |
| 65 | 4 | 50 | 195 | ROOT_S | MATR |  | 1.9038 | | IL | 0.0173 | IR | 0.0073 | MR | 0.8903 | D | 0.0852 |  |  |  |  |
| 66 | 21 | 0 | 4 | ROOT_IL | BIF |  | 1.4397 | | IL | 0.6553 | IR | 0.0445 | B | 0.3002 |  |  |  |  |  |  |
| 67 | 22 | 5 | 126 | ROOT_IL | MATP |  | 0.9402 | | IL | 0.1673 | IR | 0.1394 | MP | 0.5904 | ML | 0.0443 | MR | 0.0259 | D | 0.0327 |
| 68 | 23 | 5 | 119 | ROOT_IL | MATL |  | 0.8046 | | IL | 0.3108 | IR | 0.1936 | ML | 0.4610 | D | 0.0346 |  |  |  |  |
| 69 | 24 | 1 | 15 | ROOT_IL | MATR |  | 1.0926 | | IL | 0.1419 | IR | 0.0501 | MR | 0.6538 | D | 0.1541 |  |  |  |  |
| 70 | 26 | 0 | 31 | ROOT_IR | BIF |  | 0.9361 | | IR | 0.2827 | B | 0.7173 |  |  |  |  |  |  |  |  |
| 71 | 27 | 5 | 227 | ROOT_IR | MATP |  | 1.5494 | | IR | 0.1884 | MP | 0.7090 | ML | 0.0165 | MR | 0.0588 | D | 0.0273 |  |  |
| 72 | 28 | 4 | 701 | ROOT_IR | MATL |  | 1.6332 | | IR | 0.3681 | ML | 0.5752 | D | 0.0566 |  |  |  |  |  |  |
| 73 | 29 | 0 | 160 | ROOT_IR | MATR |  | 1.2428 | | IR | 0.2633 | MR | 0.6809 | D | 0.0558 |  |  |  |  |  |  |

Table 1: **Dirichlet priors for transitions.** “dist #”= index for the 73 different types of transition distributions in CMs; asterisks mark six distributions which had very few observed counts even after tying into groups, and for which we assigned a uniform plus-one Laplace prior. “tied to”= if an index is shown in this column, this distribution was estimated in a group (pooling observed counts) with the indicated distribution. “dist counts”= total counts observed for this distribution in the training data, before pooling into groups. “grp counts”= total counts for a group of one or more pooled distributions; this is the size of the training data sets for 36 different single-component Dirichlet priors. “node state”= unique CM state type the transition is from. “next node”= the node type that the transitions are going to. The codes for node types and state types in a CM are more fully explained in [16].

|  |  | # filtered | # aln | # consensus | # consensus | base pair | SS |
| --- | --- | --- | --- | --- | --- | --- | --- |
| alignment | # seqs | seqs | columns | base pairs | SS columns | counts | counts |
| LSU | 1551 | 139 | 7270 | 601 | 1532 | 65229 | 180558 |
| SSU bap | 12773 | 254 | 2653 | 421 | 680 | 97834 | 153565 |
| SSU euk | 7151 | 207 | 4558 | 407 | 959 | 72521 | 174260 |
| SSU mito | 1039 | 107 | 3791 | 216 | 524 | 19803 | 56510 |

Table 2: **Summary statistics for the dataset used for emission prior estimation.** “SS” = single-stranded.

| component *i* | | 1 | 2 | 3 | 4 | 5 | 6 | 7 | 8 | 9 |
| --- | --- | --- | --- | --- | --- | --- | --- | --- | --- | --- |
|  | | 0.0305 | 0.0703 | 0.1185 | 0.1810 | 0.1888 | 0.1576 | 0.0417 | 0.0959 | 0.1156 |
| \|α\| | | 14.3744 | 2.9920 | 26.2757 | 0.5342 | 4.2716 | 13.3232 | 33.8619 | 22.2258 | 33.1991 |
|  | | | | | | | | | | |
| *ab* | mean α |  |  |  |  |  |  |  |  |  |
| AA | 0.0063 | 0.0398 | 0.0390 | 0.0011 | 0.0017 | 0.0005 | 0.0062 | 0.0064 | 0.0058 | 0.0002 |
| AC | 0.0092 | 0.0421 | 0.0176 | 0.0009 | 0.0152 | 0.0018 | 0.0125 | 0.0115 | 0.0051 | 0.0046 |
| AG | 0.0052 | 0.0381 | 0.0226 | 0.0046 | 0.0034 | 0.0008 | 0.0032 | 0.0040 | 0.0053 | 0.0001 |
| AU | **0.1663** | **0.1092** | 0.0864 | 0.0194 | **0.2138** | **0.1464** | **0.2563** | **0.7360** | **0.1295** | 0.0404 |
|  |  |  |  |  |  |  |  |  |  |  |
| CA | 0.0086 | 0.0412 | 0.0510 | 0.0054 | 0.0027 | 0.0044 | 0.0018 | 0.0030 | 0.0138 | 0.0002 |
| CC | 0.0038 | 0.0327 | 0.0115 | 0.0030 | 0.0001 | 0.0003 | 0.0036 | 0.0039 | 0.0035 | 0.0041 |
| CG | **0.2412** | **0.1007** | **0.1392** | **0.8310** | **0.1359** | **0.3211** | 0.0889 | 0.0340 | **0.2870** | 0.0147 |
| CU | 0.0066 | 0.0418 | 0.0172 | 0.0027 | 0.0104 | 0.0019 | 0.0045 | 0.0076 | 0.0052 | 0.0003 |
|  |  |  |  |  |  |  |  |  |  |  |
| GA | 0.0061 | 0.0362 | 0.0266 | 0.0002 | 0.0074 | 0.0002 | 0.0058 | 0.0045 | 0.0042 | 0.0021 |
| GC | **0.2547** | **0.1299** | 0.0544 | 0.0206 | **0.1786** | **0.1613** | **0.4079** | 0.0945 | **0.1155** | **0.8858** |
| GG | 0.0063 | 0.0327 | 0.0142 | 0.0045 | 0.0091 | 0.0005 | 0.0072 | 0.0023 | 0.0044 | 0.0030 |
| GU | 0.0567 | 0.0811 | 0.0412 | 0.0049 | **0.1355** | 0.0451 | 0.0668 | 0.0303 | 0.0356 | 0.0218 |
|  |  |  |  |  |  |  |  |  |  |  |
| UA | **0.1571** | **0.1063** | **0.3085** | 0.0672 | **0.1856** | **0.2293** | 0.0902 | 0.0363 | **0.3108** | 0.0151 |
| UC | 0.0063 | 0.0477 | 0.0263 | 0.0006 | 0.0048 | 0.0002 | 0.0056 | 0.0042 | 0.0060 | 0.0038 |
| UG | 0.0543 | 0.0746 | **0.1054** | 0.0317 | 0.0807 | 0.0814 | 0.0299 | 0.0120 | 0.0551 | 0.0032 |
| UU | 0.0114 | 0.0459 | 0.0389 | 0.0022 | 0.0151 | 0.0048 | 0.0098 | 0.0095 | 0.0133 | 0.0008 |

Table 3: **Parameters of the 9 component Dirichlet mixture emission prior for base pairs.**

$q_{i}$ = mixture coefficient for component *i*. Normalized α values >0.10 are in bold faced type (0.10 was arbitrarily chosen to highlight higher values).

| component *i* | | 1 | 2 | 3 | 4 | 5 | 6 | 7 | 8 |
| --- | --- | --- | --- | --- | --- | --- | --- | --- | --- |
|  | | 0.0851 | 0.0159 | 0.1020 | 0.4160 | 0.0745 | 0.0554 | 0.1184 | 0.1327 |
| \|α\| | | 15.4467 | 154.464 | 180.286 | 5.4562 | 0.2199 | 16.4089 | 13.4592 | 19.9059 |
|  |  |  |  |  |  |  |  |  |  |
| *a* | mean α |  |  |  |  |  |  |  |  |
| A | **0.3951** | 0.0373 | **0.9961** | **0.9787** | **0.3109** | **0.3383** | 0.0375 | 0.0864 | **0.8247** |
| C | **0.1635** | 0.0490 | 0.0015 | 0.0052 | **0.2067** | **0.1782** | **0.8916** | 0.0303 | 0.0493 |
| G | **0.2041** | 0.0220 | 0.0023 | 0.0072 | **0.1751** | **0.2905** | 0.0182 | **0.8313** | 0.0569 |
| U | **0.2372** | **0.8917** | 0.0000 | 0.0090 | **0.3073** | **0.1930** | 0.0527 | 0.0519 | 0.0691 |

Table 4: **Parameters of the 8 component Dirichlet mixture emission prior for singlets.**

$q_{i}$ = mixture coefficient for component *i*. Normalized α values >0.10 are in bold faced type (0.10 was arbitrarily chosen to highlight higher values).

| Rfam 7.0 | family | # | # | avg len |  | non-banded | QDB () | | | QDB () | | | |
| --- | --- | --- | --- | --- | --- | --- | --- | --- | --- | --- | --- | --- | --- |
| ID | name | query | test | query | W | time | time | | spd up | MER | FP | FN | thr |
| RF00177 | SSU_rRNA_5 | 145 | 21 | 593 | 690 | 96.97 | 7.61 | | 12.74 | 0 | 0 | 0 | 9.90 |
| RF00024 | Telomerase-vert | 20 | 11 | 436 | 505 | 52.99 | 4.44 | | 11.94 | 0 | 0 | 0 | 11.30 |
| RF00011 | RNaseP_bact_b | 30 | 1 | 366 | 441 | 33.44 | 3.72 | | 8.98 | 0 | 0 | 0 | 11.31 |
| RF00018 | CsrB | 8 | 1 | 351 | 403 | 28.76 | 2.27 | | 12.68 | 0 | 0 | 0 | 12.98 |
| RF00040 | rne5 | 6 | 1 | 338 | 368 | 19.69 | 2.60 | | 7.57 | 0 | 0 | 0 | 11.79 |
| RF00023 | tmRNA | 19 | 40 | 334 | 463 | 24.58 | 2.47 | | 9.95 | 11 | 0 | 11 | 11.20 |
| RF00010 | RNaseP_bact_a | 233 | 1 | 332 | 514 | 33.82 | 3.29 | | 10.27 | 0 | 0 | 0 | 12.61 |
| RF00009 | RNaseP_nuc | 26 | 21 | 320 | 530 | 27.89 | 7.11 | | 3.93 | 19 | 0 | 19 | 11.67 |
| RF00017 | SRP_euk_arch | 28 | 21 | 303 | 328 | 14.78 | 2.70 | | 5.48 | 6 | 0 | 6 | 10.40 |
| RF00028 | Intron_gpI | 5 | 24 | 300 | 381 | 17.55 | 3.59 | | 4.88 | 19 | 2 | 17 | 10.70 |
| RF00373 | RNaseP_arch | 20 | 13 | 290 | 337 | 15.06 | 3.12 | | 4.82 | 0 | 0 | 0 | 12.23 |
| RF00030 | RNase_MRP | 18 | 3 | 284 | 394 | 19.75 | 3.12 | | 6.34 | 3 | 0 | 3 | 12.46 |
| RF00101 | SraC_RyeA | 6 | 1 | 250 | 278 | 9.50 | 1.54 | | 6.19 | 0 | 0 | 0 | 11.88 |
| RF00230 | T-box | 10 | 35 | 244 | 298 | 8.23 | 1.80 | | 4.58 | 1 | 0 | 1 | 12.34 |
| RF00448 | IRES_EBNA | 7 | 1 | 213 | 238 | 7.25 | 1.26 | | 5.76 | 1 | 0 | 1 | 11.99 |
| RF00012 | U3 | 6 | 5 | 212 | 240 | 7.17 | 1.61 | | 4.46 | 2 | 0 | 2 | 13.02 |
| RF00174 | Cobalamin | 87 | 66 | 203 | 326 | 9.49 | 2.85 | | 3.32 | 0 | 0 | 0 | 11.28 |
| RF00004 | U2 | 76 | 1 | 184 | 215 | 5.95 | 1.12 | | 5.29 | 0 | 0 | 0 | 10.01 |
| RF00234 | glmS | 8 | 3 | 181 | 303 | 7.98 | 1.83 | | 4.36 | 0 | 0 | 0 | 11.22 |
| RF00168 | Lysine | 33 | 17 | 180 | 223 | 5.71 | 1.45 | | 3.94 | 0 | 0 | 0 | 15.98 |
| RF00380 | ykoK | 35 | 3 | 168 | 192 | 4.33 | 1.23 | | 3.52 | 0 | 0 | 0 | 13.10 |
| RF00003 | U1 | 46 | 6 | 159 | 184 | 4.14 | 0.89 | | 4.63 | 0 | 0 | 0 | 11.24 |
| RF00025 | Telomerase-cil | 10 | 2 | 157 | 188 | 3.89 | 1.00 | | 3.88 | 2 | 0 | 2 | 13.97 |
| RF00002 | 5_8S_rRNA | 62 | 1 | 151 | 183 | 3.44 | 0.97 | | 3.55 | 0 | 0 | 0 | 11.28 |
| RF00379 | ydaO-yuaA | 31 | 4 | 147 | 227 | 4.38 | 1.63 | | 2.69 | 0 | 0 | 0 | 12.25 |
| RF00067 | U15 | 9 | 3 | 146 | 178 | 2.71 | 0.98 | | 2.76 | 0 | 0 | 0 | 11.11 |
| RF00029 | Intron_gpII | 7 | 11 | 141 | 276 | 4.75 | 1.32 | | 3.59 | 1 | 0 | 1 | 11.08 |
| RF00015 | U4 | 25 | 1 | 141 | 187 | 3.66 | 1.04 | | 3.53 | 1 | 0 | 1 | 13.46 |
| RF00096 | U8 | 5 | 1 | 135 | 177 | 2.98 | 0.93 | | 3.20 | 0 | 0 | 0 | 11.56 |
| RF00080 | yybP-ykoY | 20 | 33 | 129 | 173 | 3.05 | 1.13 | | 2.69 | 1 | 0 | 1 | 10.78 |
| RF00114 | S15 | 10 | 1 | 117 | 138 | 1.78 | 0.60 | | 2.99 | 0 | 0 | 0 | 13.12 |
| RF00020 | U5 | 29 | 3 | 115 | 139 | 2.06 | 0.74 | | 2.80 | 0 | 0 | 0 | 13.64 |
| RF00059 | THI | 228 | 8 | 109 | 222 | 3.34 | 1.46 | | 2.29 | 0 | 0 | 0 | 13.66 |
| RF00504 | gcvT | 109 | 5 | 102 | 199 | 2.37 | 1.40 | | 1.70 | 0 | 0 | 0 | 13.40 |
| RF00167 | Purine | 33 | 4 | 99 | 119 | 1.49 | 0.57 | | 2.62 | 0 | 0 | 0 | 13.02 |
| RF00169 | SRP_bact | 46 | 15 | 96 | 120 | 1.47 | 0.65 | | 2.26 | 0 | 0 | 0 | 11.58 |
| RF00055 | snoZ37 | 5 | 1 | 94 | 117 | 1.14 | 0.53 | | 2.16 | 1 | 0 | 1 | 13.96 |
| RF00019 | Y | 15 | 1 | 94 | 128 | 1.42 | 0.73 | | 1.94 | 1 | 0 | 1 | 14.25 |
| RF00033 | MicF | 8 | 1 | 93 | 114 | 1.28 | 0.51 | | 2.50 | 0 | 0 | 0 | 13.18 |
| RF00213 | snoR38 | 7 | 3 | 88 | 147 | 1.36 | 0.70 | | 1.94 | 0 | 0 | 0 | 16.07 |
| RF00054 | U25 | 5 | 1 | 87 | 107 | 0.96 | 0.46 | | 2.09 | 1 | 0 | 1 | 16.66 |
| RF00206 | U54 | 12 | 1 | 81 | 115 | 0.94 | 0.53 | | 1.76 | 1 | 0 | 1 | 15.80 |
| RF00104 | mir-10 | 9 | 2 | 73 | 94 | 0.85 | 0.52 | | 1.64 | 2 | 0 | 2 | 16.13 |
| RF00005 | tRNA | 1080 | 19 | 73 | 127 | 1.35 | 0.48 | | 2.81 | 5 | 1 | 4 | 12.62 |
| RF00170 | msr | 5 | 3 | 70 | 112 | 0.86 | 0.45 | | 1.92 | 3 | 0 | 3 | 13.49 |
| RF00163 | Hammerhead_1 | 65 | 1 | 68 | 233 | 1.60 | 0.90 | | 1.77 | 0 | 0 | 0 | 15.82 |
| RF00031 | SECIS | 11 | 24 | 64 | 87 | 0.69 | 0.42 | | 1.63 | 13 | 2 | 11 | 14.58 |
| RF00165 | Corona_pk3 | 10 | 1 | 63 | 80 | 0.55 | 0.31 | | 1.78 | 1 | 0 | 1 | 14.72 |
| RF00066 | U7 | 28 | 2 | 62 | 85 | 0.59 | 0.34 | | 1.73 | 0 | 0 | 0 | 14.23 |
| RF00008 | Hammerhead_3 | 82 | 1 | 55 | 101 | 0.71 | 0.44 | | 1.61 | 0 | 0 | 0 | 14.71 |
| RF00037 | IRE | 36 | 1 | 28 | 45 | 0.17 | 0.12 | | 1.39 | 1 | 0 | 1 | 17.64 |
| MER statistics summed across all families | | | | | |  | | | | 96 | 5 | 91 | N/A |
| Summary MER stats (using one threshold for all families) | | | | | |  | | | | 114 | 3 | 111 | 16.38 |
| average timing statistics | | | | | | 10.02 | 1.64 | 4.21 | |  | | | |
| total timing statistics | | | | | | 510.86 | 83.48 | 6.12 | |  | | | |

Table 5: **Rfam benchmark families with timing and MER statistics.** “W” = window length, maximum size of a hit per family, calculated as dmax(0). Running times for standard (non-banded) and QDB () searches are given for each family, in CPU-hours per Mb. The MER threshold (“thr” column) is the bit score for a given family at which the sum of false positives (“FP”) and false negatives (“FN”) is minimized. “MER” = minimum error rate, FP+FN at threshold. In the row labeled “Summary MER statistics”, these are derived from a single score threshold in a ranked list of all hits across all families. All statistics are for infernal version 0.72 in local alignment mode.

|  |  | entropy |  | summary | family-specific |
| --- | --- | --- | --- | --- | --- |
| program | prior | (bits) | β | MER | MER |
| blastn | - | - | - | 216 | 188 |
| infernal 0.55 | plus-1 | - | - | 232 | 180 |
| infernal 0.72 | plus-1 | - | - | 215 | 187 |
| infernal 0.72 | plus-1 | 1.46 | - | 208 | 191 |
| infernal 0.72 | informative | - | - | 177 | 158 |
| infernal 0.72 | informative | 1.46 | - | 105 | 90 |
| infernal 0.72 | informative | 1.46 |  | 114 | 96 |

Table 6: **Rfam benchmark MER summary statistics.** “prior”: “plus-1” if uninformative Laplace plus-1 priors were used; “informative” if new Dirichlet priors were used. “entropy”: target model entropy in bits for entropy weighting; “-” if entropy weighting was not used. “β”: tail probability loss for banded calculation used; “-” if search was non-banded. “summary MER”: MER across 51 benchmark families; “family-specific MER”: MER for each family, summed over all 51 families. Program versions: Row 1: WU-BLASTN-2.0MP –kap -W=7. For infernal version 0.55, window length values (*W*) were preset as calculated in version 0.72 with plus-1 priors.

Figure 1: **An example RNA family and corresponding CM.** **(A):** A toy multiple alignment of three RNA sequences, with 28 total columns, 24 of which will be modeled as consensus positions. The [structure] line annotates the consensus secondary structure: < and > symbols mark base pairs, :’s mark consensus single stranded positions, and .’s mark “insert” columns that will not be considered part of the consensus model because more than half the sequences in these columns contain gaps. **(B):** The structure of one sequence from (A), the same structure with positions numbered according to alignment columns, and the guide tree of nodes corresponding to that structure, with alignment column indices assigned to nodes (for example, node 5, a MATP “match-pair” node, will model the consensus base pair between columns 4 and 14). **(C):** The state topology of three selected nodes of the CM, for two MATP nodes and one consensus “leftwise” single residue bulge node (MATL, “match-left”). The consensus pair and singlet states (two MPs and one ML) are white, and the insertion/deletion states are grey. State transitions are indicated by arrows.

Figure 2: **Effect of transition priors on band calculation.** Predicted and actual target lengths are shown for three CMs built from alignments of five tRNA, 5S rRNA, and RNaseP sequences, which are about 75, 120, and 380 residues long, respectively. Solid vertical lines are histogram bars of the actual lengths of the query sequences in each alignment, corresponding with the right vertical axis labels. Dashed and dotted curves show QDB calculations for $\gamma_{0}(d)$ for the root state of each model, for uninformative versus informative Dirichlet priors, respectively. Dashed and dotted vertical lines show the band bounds (dmin(0) (left) and dmax(0) (right)) derived from the $\gamma_{0}(d)$ distributions using . The uninformative plus-one prior results in consistent underprediction of target sequence lengths, with a broad distribution. The new informative priors produce tighter distributions that are centered on the actual subsequence lengths. We observe the same result for all other states (data not shown).

Figure 3: **Effect of varying the β parameter on sensitivity, specificity, and speedup.**

Figure 4: **CPU time required by CM searches with and without QDB.** The time required for searching the 1 Mb target pseudogenome with each of the 51 benchmark models is shown as a point, plotted on a log-log graph as a function of the average length of the RNA sequences in the query alignment; open circles are without QDB, and filled circles are with QDB (with the default $\beta=10^{-7}$). Lines represent fits to a power law (), showing that for a fixed *L*= 1 Mb target database size, the standard CYK algorithm empirically scales as and the QDB algorithm scales as . The apparent intersection of the linear fitted lines is deceptive. At small query lengths, run time is dominated by factors other than the CM alignment computation, such as i/o. QDB searches are always faster than non-banded searches even for synthetic tiny queries of less than 10 nt (data not shown).

Figure 5: **ROC curves for the benchmark.** Plots are shown for the new infernal 0.72 with and without QDB, for the old infernal 0.55, and for family-pairwise-searches (FPS) with blastn.
